# Granulocyte-Macrophage Colony Stimulating Factor Receptor Contributes to Plexiform Neurofibroma Initiation

**DOI:** 10.3390/cancers17050905

**Published:** 2025-03-06

**Authors:** Jay Pundavela, Ashley Hall, Samantha Anne Dinglasan, Kwangmin Choi, Tilat A. Rizvi, Bruce C. Trapnell, Jianqiang Wu, Nancy Ratner

**Affiliations:** 1 Division of Experimental Hematology and Cancer Biology, Cincinnati Children’s Hospital Medical Center, Cincinnati, OH 45229, USA; jay.pundavela@cchmc.org (J.P.); ashley.hall@cchmc.org (A.H.); samanthaanne.dinglasan@cchmc.org (S.A.D.); kwangmin.choi@cchmc.org (K.C.); tilat.rizvi@cchmc.org (T.A.R.); jianqiang.wu@cchmc.org (J.W.); 2 Translational Pulmonary Science Center, Cincinnati Children’s Hospital Medical Center, Cincinnati, OH 45229, USA; bruce.trapnell@cchmc.org; 3 Departments of Medicine and Pediatrics, College of Medicine, University of Cincinnati, Cincinnati, OH 45267, USA; 4 Department of Pediatric, College of Medicine, University of Cincinnati, Cincinnati, OH 45229, USA

**Keywords:** Neurofibromatosis Type 1, NF1, neurofibroma, GM-CSFR-α, GM-CSFR-β^c^, macrophage, dendritic cells

## Abstract

Plexiform neurofibroma (PNF) is an immune cell-rich peripheral nerve sheath tumor that develops primarily in individuals with Neurofibromatosis Type 1 (NF1). Granulocyte-macrophage colony stimulating factor receptor-β (GM-CSFR-β^c^) is a shared component of receptors for the cytokines GM-CSF, IL-3, and IL-5, ligands with immunomodulatory and tumor promoting roles. In the present study, we use genetically engineered mouse model of neurofibroma. We identified the expression of GM-CSFR-β^c^ and GM-CSFR-α on PNF cells and on macrophages and dendritic cells in the PNF, using the Nf1^f/f^; DhhCre mouse model of neurofibroma formation. Genetic deletion of GM-CSFR-β^c^ in this model reduced the number of PNFs, which was associated with decreased numbers of tumor-associated Iba1+ macrophages and CD11c+ dendritic cells (DC), while loss of GM-CSFR-α had no effect. Deletion of GM-CSFR-α or GM-CSFR-β^c^ did not improve mouse survival or the structure of Remak bundles in peripheral nerves. Proteome analysis of tumor lysates showed altered levels of numerous cytokines after receptor loss, suggesting that the compensatory effects of other cyto/chemokines maintain a proinflammatory environment promoting neurofibroma. Thus, GM-CSFR-β^c^ signaling contributes modestly to neurofibroma formation, apparently independently of its ligand GM-CSF.

## Simple Summary

Granulocyte-macrophage colony stimulating factor (GM-CSF) is a cytokine known for its role in regulating inflammation and tumorigenesis. GM-CSF binds the alpha (GM-CSFR-α) and beta-common subunit (GM-CSFR-β^c^) receptor complex. Plexiform neurofibroma (PNF) is an NF1-associated nerve tumor. In a PNF mouse model (Nf1^f/f^; DhhCre), we found that PNF cells show an increase in GM-CSF gene expression and that GM-CSFR-α and GM-CSFR-β^c^ are expressed only by immune cells. Deletion of GM-CSFR-β^c^, but not GM-CSFR-α, reduced PNF numbers and the numbers of dendritic cells and macrophages. This unexpected finding suggests that other ligands that bind to GM-CSFR-β^c^ might play roles in neurofibroma. These results support a role for GM-CSFR-β^c^-mediated signaling in PNF and emphasize the plasticity of the PNF immune microenvironment.

## 1. Introduction

Granulocyte colony-stimulating factor (GM-CSF) is a cytokine encoded by the CSF2 gene. GM-CSF binds a unique receptor alpha (α) subunit GMCSFR-α and shares a beta subunit (b-common (βc)) with α receptors specific for interleukin 3 (IL-3) and interleukin 5 (IL-5) [1]. GM-CSF, as the name indicates, is known to stimulate the proliferation and differentiation/polarization of granulocytes and macrophages from bone marrow (BM) progenitor cells ex vivo [2,3,4]. Activation of the GM-CSF receptor triggers downstream signaling pathways, including the JAK2/STAT5, PI3K/AKT and MEK/ERK pathways [5]. Extensive studies of GM-CSF have demonstrated its role in promoting inflammation [5,6]. In addition, GM-CSF can either suppress or stimulate tumorigenesis, using immune and non-immune mechanisms [7].

Therapeutic strategies that seek to increase or diminish GM-CSF function are therefore being developed for use in oncology [7,8]. For example, GM-CSF can stimulate the production, recruitment and activation of antigen-presenting cells (APCs) such as macrophages and DCs to elicit a T-cell mediated anti-tumor response. Thus, oncolytic viruses containing GM-CSF and a GM-CSF-based vaccine are currently being used to treat melanoma, non-small cell lung carcinoma and prostate cancer. Recombinant human GM-CSF is used to treat blood cancers (ALL and AML) [9] and solid cancers including ovarian [10] and colorectal cancer [11,12]. In contrast, other tumors upregulate GM-CSF and its receptors to promote its survival and growth. In this setting, blockade of GM-CSF can evoke an anti-cancer response by directly killing cancer cells or by inhibiting angiogenesis and epithelial–mesenchymal transformation [13,14,15]. 

Individuals with the genetic condition Neurofibromatosis Type 1 (NF1) are predisposed to a set of diverse manifestations that include brain and peripheral nerve tumors and cognitive dysfunction. The NF1 protein, neurofibromin, is an off signal (a GTPase-activating protein; GAP) for RAS signaling proteins. Therefore, loss of NF1 results in increased and/or prolonged signaling downstream of specific receptors [16,17,18,19,20]. A rare manifestation in children with NF1 is the development of juvenile myelomonocytic leukemia (JMML), which can be mimicked in mice by deletion of *Nf1* in hematopoietic cells [21,22]. Notably, Nf1 mutant haemopoietic cells are hyper-responsive to exogenous GM-CSF, which causes myeloid cells expand; when hematopoietic cells lack Nf1 and mice are Gmcsf-/-, or Gmcsfr-βc-/-, myeloid cell numbers are reduced to wild type levels [23,24,25,26,27]. These data strongly implicate Nf1 signaling downstream of GM-CSFR activation in JMML.

Plexiform neurofibroma (PNF) is a peripheral nerve tumor that develops in at least half of individuals with NF1. Consistent with the designation of NF1 as a tumor suppressor gene, in PNF, Schwann cells show loss of function of both NF1 alleles [28]. In mice, PNF formation occurs uniquely in Schwann cells upon complete loss of *Nf1* [29,30]. Inflammation is a prominent feature of the PNF tumor microenvironment; large numbers of macrophages accumulate together with other immune cells (mast cells, T cells and dendritic cells (DCs)) to support PNF development and tumor growth [31,32,33,34,35,36]. In the Nf1^f/f^; DhhCre model, PNF cells showed increased GM-CSFR-β^c^ mRNA expression [37]. Furthermore, deletion of β^c^ attenuated nerve injury-induced pigmentation in Nf1+/- mice [38], and nerve injury itself can cause PNF formation [39]. Together, these data suggest the possibility that one or more GM-CSFR-β^c^ dependent signals might influence PNF formation or growth.

The objective of this study was to determine if GM-CSF signaling is involved in the regulation of the PNF proinflammatory environment by targeting its receptors GM-CSFR-α and GM-CSFR-β^c^. We showed that deletion of GM-CSFR-β^c^, but not GM-CSFR-α, reduced the number of PNFs and reduced the numbers of dendritic cells and macrophages. Deletion of neither receptor rescued the Remak Schwann cell pathology that is a feature of this model, nor did it significantly reduce the numbers of mast cells and T cells. Also, deletion of GM-CSFR-β^c^ in Nf1^f/f^; DhhCre mice resulted in differential expression of inflammatory PNF cytokines. These results support a role for GM-CSFR-β^c^-mediated signaling in PNF and emphasize the plasticity of the PNF immune microenvironment.

## 2. Materials and Methods

### 2.1. Mice

GM-CSFR-α and the GM-CSFR β^c^ knockout mice on a C57BL/6 background were a kind gift from Bruce C. Trapnell (CCHMC). Mouse genotyping and recombination assays were carried out as described [29,40,41]. Briefly, mouse ear or tail clips were subjected to NaOH/Tris-HCl genomic DNA extraction followed by standard polymerase chain reaction (PCR) using primers for Gm-csfrα: Primer #1 (VS4114) = CATCACATGCCATGAACATCACC; Primer #2 (VS4115) = ACCGGAAGTGACATCATTGCG. Gm-csfrβ: Primer #1 (neoF) = ATATTGCTG AAGAGCTTG GCGGC; Primer #2 (823) = GTGTAGACACTGGCCCCCG; Primer #3 (891) = GAACCTTCAATGCTTCTTTGATGGGAT. The finished PCR reaction was electrophoresed on a 2% TBE agarose gel and imaged under a c50 imaging system (Azure biosystems, Dublin, CA, USA).

### 2.2. Mouse Dissection and Tumor Number/Size Measurements

To quantify the tumor number and size, whole body dissection was performed under a Leica dissecting microscope to carefully dissect the spinal cord with the DRG/nerve roots intact, exposing the spinal cord and dorsal root ganglia (DRG) in 7-month-old mice. Image snapshots of dissected spinal cords with the attached nerves were taken and analyzed using ImageJ. (V.1.53k). A DRG with a diameter >1 mm, measured parallel to the spinal axis, was defined as a tumor.

### 2.3. Immunofluorescence (IF) and Immunohistochemistry (IHC)

Mice were cardiac-perfused with ice-cold phosphate buffer saline (PBS) (0.1 M, pH 7.4), then perfusion-fixed with pre-chilled 4% (*v*/*v*) paraformaldehyde in 1× PBS. Tissues (PNF or DRG) were postfixed overnight at 4 °C, then incubated overnight in 20% sucrose at 4 °C before embedding in optimal cutting temperature (OCT) compound (Richard-Allan Scientific, Kalamazoo, MI, USA) for cryosectioning. Frozen sections (10 μm each) were blocked with 10% serum in 0.3% Triton-X in 1× PBS for 1 h at room temperature then single or co-labeled with primary antibodies overnight at 4 °C followed by fluorescently labeled secondary goat antibodies. The following primary antibodies were used: anti-CD3 (17A2], (BioLegend, San Diego, CA, USA), anti-CD11c (N418), Thermo Fisher Scientific, Waltham, MA, USA), anti-CD45 ((I3/2.3), Abcam, Cambridge, UK), and anti-Iba-1 (019-19741, Wako, Tokyo, Japan) in blocking solution. Primary antibody binding was visualized with appropriate combinations of anti-rabbit (Alexa Fluor 488, A-11034, Invitrogen, Waltham, MA, USA), anti-rat (Alexa Fluor 555, A-21434, Invitrogen), or anti-hamster (Alexa Fluor 647, 127-605-160, Jackson ImmunoResearch, West Grove, PA, USA) secondary antibodies for 1 h. Finally, cell nuclei were labeled with DAPI (0.1 μg/mL), and slides were mounted with cover glasses using Fluoromount G (Electron Microscopy Sciences, Hatfield, PA, USA). Tris-buffered saline with Tween 20 (TBST) rinses were performed between steps.

For immunohistochemistry (IHC), the procedure was as for immunofluorescence (IF), except that following overnight incubation with anti-GM-CSFR beta rabbit polyclonal antibody (Bioss; cat# bs-3689R, Woburn, MA, USA) or anti-GM-CSFR alpha rabbit polyclonal antibody (Bioss; cat# bs-1457R, USA) at 4 °C, the sections were subsequently incubated with biotinylated goat anti-rabbit secondary antibody (VectorLabs; cat#BA-1000, Newark, CA, USA) for 1 h and rinsed three times in TBST for 5 min each. After incubating with avidin-biotin complex (ABC) (VectorLabs; cat#PK-6100, USA) for 1 h and rinsing in TBST, the sections were incubated in 3,3′-diaminobenzidine (DAB) (VectorLabs; cat#SK-4100, USA) for 5 min, then rinsed, dehydrated, and mounted with Histomount (Life Technologies, Carlsbad, CA, USA).

### 2.4. Tumor/DRG Processing and Flow Cytometry Analysis

Paraspinal tumors or dorsal root ganglia (DRG) samples were prepared for flow cytometry analysis as in Pundavela et al. [42]. In brief, tumors or DRG/nerves were dissected from mice perfused with ice-cold PBS, minced, and subjected to enzymatic and mechanical dissociation. After filtering the cell suspension through 100 µm and 70 µm strainers, flow cytometry was performed, using autologous spleen or beads as controls. The following antibodies were utilized: anti-GMCSF alpha Alexa Fluor^®^ 647 anti-CD116 (EPR24554-26, ab283228, Abcam, Boston, MA, USA), mouse anti-GMCSF beta IL-3R beta Alexa Fluor^®^ 594 (FAB5492T-100UG, R&D Systems, Minneapolis, MN, USA), anti-TCRβ (cat# 109243, Biolegend, San Diego, CA, USA), anti-CD8a PerCP, CD4 BUV395 (cat# 563790, BD Horizon, Franklin Lakes, NJ, USA), anti-CD11b (plate_number_2, cat# 101222, Biolegend), and several others. Samples were analyzed with the Aurora cell multiparametric flow analyzer (Cytek Biosciences, Fremont, CA, USA) and FlowJO_V10 or SpectroFlo 3.3.0 software.

### 2.5. Mouse Cytokine Array Analysis

Mouse cytokine protein expressions were quantified using a mouse cytokine array (Proteome Profiler Mouse XL Cytokine Array, R&D Systems, Minneapolis, MN, USA). In brief, protein lysates were prepared from the paraspinal tumors of Nf1^f/f^; DhhCre mouse neurofibromas, which exhibited a global deletion of either GM-CSFR-α or GM-CSFR-βc. The arrays were conducted according to the instructions provided by R&D Systems, utilizing 400 μg of lysate protein. The intensities of the dots were measured and quantified using the Protein Array Analyzer tools in ImageJ software (v.1.43k).

### 2.6. Electron Microscopy

Electron microscopy was carried out as described [42,43]. In summary, mice were perfused with a fixative solution consisting of 4% *v*/*v* paraformaldehyde (PFA) and 2.5% glutaraldehyde in 0.1 M phosphate buffer saline (PBS) at pH 7.4. The saphenous nerve was then dissected and post-fixed overnight, transferred to 0.175 M cacodylate buffer, osmicated, dehydrated, and embedded in Embed 812 (Ladd Research Industries, Williston, VT, USA). Ultrathin sections were stained with uranyl acetate and lead citrate and observed using a Hitachi H-7600 electron microscope, Hitachi High-Tech Corp., Tokyo, Japan.

### 2.7. Statistical Analysis

Survival data were compared using Kaplan–Meyer analysis and a log-rank (Mantel–Cox) test. Other data were analyzed using two-tailed Student’s *t*-tests, one-way or two-way ANOVA with multiple comparisons as appropriate. A *p*-value < 0.05 is considered significant. Generation of graphs and statistical analyses were performed using GraphPad Prism v.10.

## 3. Results

### 3.1. GM-CSFR-α and GM-CSFR-β^c^ Are Expressed by PNF Macrophages and Dendritic Cells

**Figure 1:**
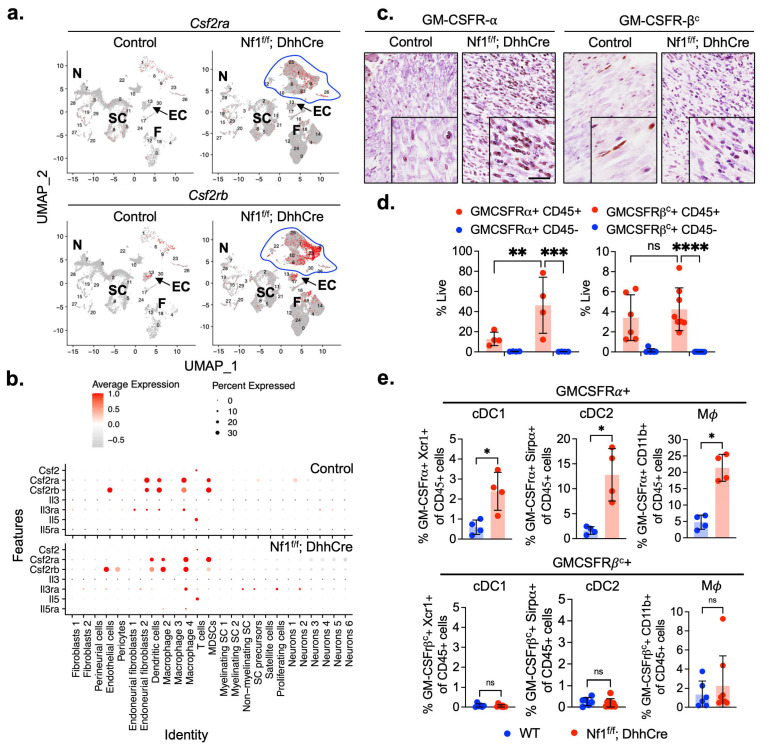
Gene and protein expression of GM-CSF alpha (GM-CSFR-α) and beta-common (GM-CSFR-β^c^) receptors in 7-month-old mice. (**a**) UMAP of PNF from 7-month-old Nf1^f/f^; DhhCre mice compared to aged-match wild-type (WT) control DRG. (**b**) Dot plot showing gene expression in various cell types (y-axis: Identity). (**c**) Representative pictures of immunostaining for GM-CSFR-α and GM-CSFR-β^c^ in WT DRG and PNF tissue sections. (**d**) Frequency of non-immune (CD45-) and immune (CD45+) cells in WT DRG (n = 6) and PNF (n = 8)and (**e**) subtypes of CD45+ immune cells expressing GM-CSFR-α ((WT DRG (n = 4) and PNF (n = 4)) and GM-CSFR-β^c^ ((WT DRG (n = 6) and PNF (n = 8)).Unpaired *t*-test or 2-way ANOVA multiple comparison test, * *p* < 0.05, ** *p* < 0.001, *** *p* < 0.0001, **** *p* < 0.00001.

In a previous single-cell RNA-seq dataset (GSE181985), cells from normal paraspinal cervical dorsal root ganglia (DRG)/nerves were compared to cells from PNFs (Nf1^f/f^; DhhCre) that developed in this region. In that study, we noted that *Csf2rb* was a marker of macrophage clusters that expanded when tumors developed [34]. We re-analyzed the expression pattern in additional detail, using a UMAP representation of the same data (Figure 1a). Thus, we analyzed: gene expression of *Csf2*, encoding GM-CSF; *Csf2rb*, encoding GM-CSFR-β^c^; transcriptional upregulation of *Csf2ra*, encoding GM-CSFR-α; and *Csf2rb*, encoding GM-CSFR β^c^. These genes were detected largely in immune cell clusters (clusters 6, 9, and 23) (enclosed blue line) and not in Schwann cells (clusters 5, 11, and 21) (SC) or neurons (clusters 3, 7, and 28) (N). *Csf2rb* was also expressed by PNF endothelial cells (cluster 13, arrows) and some fibroblasts (cluster 18) (F) compared to controls. Dot plot analysis of expression in each cluster from single cell RNA-Req was used to predict which cells expressed receptors (*Csf2ra*, *Il5ra*, *Il3a*, and *Csf2rb*) and their ligands (*Csf2*, *Il3,* and *Il5*) (Figure 1b). All macrophage and dendritic cell subpopulations showed *Csf2ra* and *Csf2rb* receptor expression. *Il5ra* and *Il3ra* were detected in fewer cell types in immune and non-immune cells. *Csf2* and *Il5* were present uniquely in T cells.

We conducted immunohistochemistry to definitively assess the expression of proteins encoded by these mRNAs. The results showed that GM-CSFR-α immune-positive cells were significantly enriched in the PNF compared to control DRG/nerves. In contrast, GM-CSFR-βc positive cells were present in relatively similar quantities across both groups (Figure 1c). We validated these results using flow cytometric analysis. GM-CSFR-α and GM-CSFR-β^c^ were detectable only among CD45+ immune cells, and GM-CSFR-α expressing cells were increased in PNFs versus control cells (Figure 1d). Thus, both methods showed an increase in GM-CSFR-α expressing cells in PNFs versus control cells, and GM-CSFR-β^c^ was present in a similar percentage of cells. 

Flow cytometry was next used to identify the major types of immune cells expressing these receptors. We identified significant increases in GM-CSFR-α expressing conventional dendritic cells (Xcr1+ cDC1 and Serpa+ cDC2) in Cd11b+; CD11c- macrophages (Mϕ). There was no difference in the percentages of these GM-CSFR-β^c^ expressing immune cell subtypes between PNF and WT control DRG/nerves (Figure 1e). Thus, *Csfr2a*/Gmcsfr-α and *Csfr2b*/Gmcsfr-βc are the predominant mRNAs detected by single cell analysis in PNF, showing expression mainly by immune cells in PNF. Their abundance underscores their crucial role in the immune response within this context. Other α receptors (*Il3ra* and *Il5ra*) and the ligand *Il5* are predicted to be present as shown in the dot plot in Figure 1b, but with lower expression and in fewer cells or cell types. Therefore, we focused on Csfr2a and Csfr2b.

### 3.2. Csfr2b/Gmcsfr/βc Loss Reduces Neurofibroma Numbers in Nf1^f/f^; DhhCre Mice

To identify potential roles of these receptors in PNF, we crossed Nf1^f/f^; DhhCre with mice bearing the deletion of Csfr2a (GM-CSFR-α-/-; Nf1^f/f^; DhhCre) or Csfr2b (GM-CSFR-β^c^-/-; Nf1^f/f^; DhhCre) receptors (Supplemental Figure S1). GM-CSFR-α-/- and GM-CSFR-β^c^-/- mice have been characterized [40,41,44,45]. These mice show pathology, largely lung lymphocyte patches and lung proteinuria that is caused by macrophage abnormalities and is characterized by periodic acid–Schiff positive (PAS+) protein accumulation. We confirmed PAS+ accumulation in our double mutant mice (Supplemental Figure S2). Subsets of myeloid cells are abnormal in the GM-CSFR-α-/- and GM-CSFR-βc-/- mice, so we cannot exclude potential confounding effects of these abnormal cells on the neurofibromas. However, as GM-CSFR-βc-/- mutants rescue abnormalities in the setting of a *Nf1-/-* JMML model, we wondered if we might find a similar rescue in the neurofibroma model.

**Figure 2:**
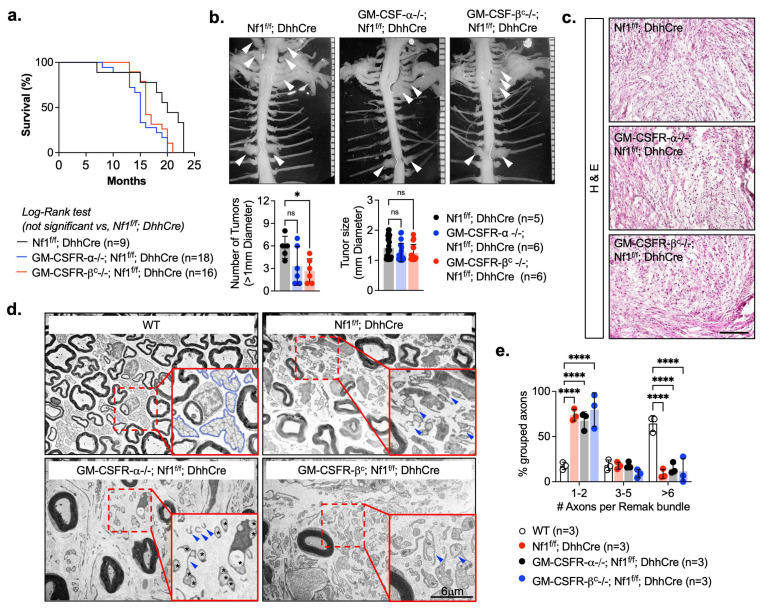
Effects of GM-CSFR-α and GM-CSFR β^c^ genetic deletion on survival and nerve pathology. (**a**) Kaplan–Meier survival curve of Nf1^f/f^; DhhCre (control) (n = 9) and mice bearing deletion of the receptor GM-CSFR-α (GM-CSFR-α-/-; Nf1^f/f^; DhhCre (n = 18)) or GM-CSFR-β^c^ (GM-CSFR-β^c^-/-; Nf1^f/f^; DhhCre (n = 16)). (**b**) representative pictures of aged-matched gross dissection of spinal cord from each mouse (Nf1^f/f^; DhhCre (control) (n = 5), GM-CSFR-α-/-; Nf1^f/f^; DhhCre (n = 6) and GM-CSFR-β^c^-/-; Nf1^f/f^; DhhCre (n = 6)) and quantification of tumor number and size (diameter). White arrows indicate PNF. (**c**) Representative images of tissue sections stained with H&E and (**d**) electromicrographs of saphenous nerve showing the ultrastructure of an intact Schwann cell Remak bundle (blue margin) in WT mice compared to the disrupted Remak structure (blue arrows) in tumor mice in the presence or loss of GM-CSF receptors. (**e**) High percentage (6 or more) of grouped axons indicate a normal Remak bundle. Two-way ANOVA multiple comparison test, * *p* < 0.05, **** *p* < 0.00001.

Homozygous deletion of Csfr2a (n = 20) or Csfr2b (n = 20) receptors did not significantly affect survival, as judged by Kaplan–Meier analysis, compared to Nf1^f/f^; DhhCre controls (n = 10) in the same genetic background (Figure 2a). Morbidities that required animal sacrifice were: GM-CSFR-α-/-; Nf1^f/f^; DhhCre ((abscess (n = 5), sickly or lethargic (n = 3), dermatitis (n = 1)); GM-CSFR-β^c^-/-; Nf1^f/f^; DhhCre((abscess (n = 2), sickly and lethargic (n = 2)]; Nf1^f/f^; DhhCre controls [abscess (n = 5)). The prevalence of skin abscesses is consistent with the subcutaneous pre-cutaneous neurofibromas reported in this animal model, which cause skin irritation [29]. 

To examine the effects of GM-CSFR-α or GM-CSFR-β^c^ receptor loss on paraspinal tumors, 7-month-old mice GM-CSFR-α-/-; Nf1^f/f^; DhhCre (n = 5); GM-CSFR-β^c^-/-; Nf1^f/f^; DhhCre (n = 5); and Nf1^f/f^; DhhCre controls (n = 5) were subjected to whole body dissection. Gross images of spinal cord/DRG peripheral nerves are shown in Figure 2b. Mice with the loss of GM-CSFR-α showed no significant reduction of tumor number. In contrast, the absence of GM-CSFR-β^c^ significantly decreased the tumor number compared to Nf1^f/f^; DhhCre controls, but the size of the tumors was not significantly changed. These results suggest a role in tumor initiation rather than tumor growth.

Spinal nerve hypertrophy with increased cellularity is a pathohistological feature of the Nf1^f/f^; DhhCre model [1]. Deletion of GM-CSFR-α or GM-CSFR-β^c^ in Nf1^f/f^; DhhCre mice did not affect the pathohistological characteristics of the tissue sections from all tumors (Figure 2c). In normal nerves, multiple small axons are grouped together by non-myelinating Schwann cells in packets called Remak bundles. Axons become progressively fewer in each Remak bundle in mouse models of neurofibroma, and the bundles are disrupted in human neurofibromas [29,46,47,48,49]. We analyzed nerve ultrastructure by electron microscopy to define the morphology of Remak bundles using previously described methods [42,50]. We found that disruption of the Nf1^f/f^; DhhCre mice Remak bundles was not rescued by the absence of GM-CSFR-α or GM-CSFR β^c^ (Figure 2d,e).

### 3.3. Deletion of GM-CSFR-βc Reduces Myeloid Cell Infiltrates in PNF

**Figure 3:**
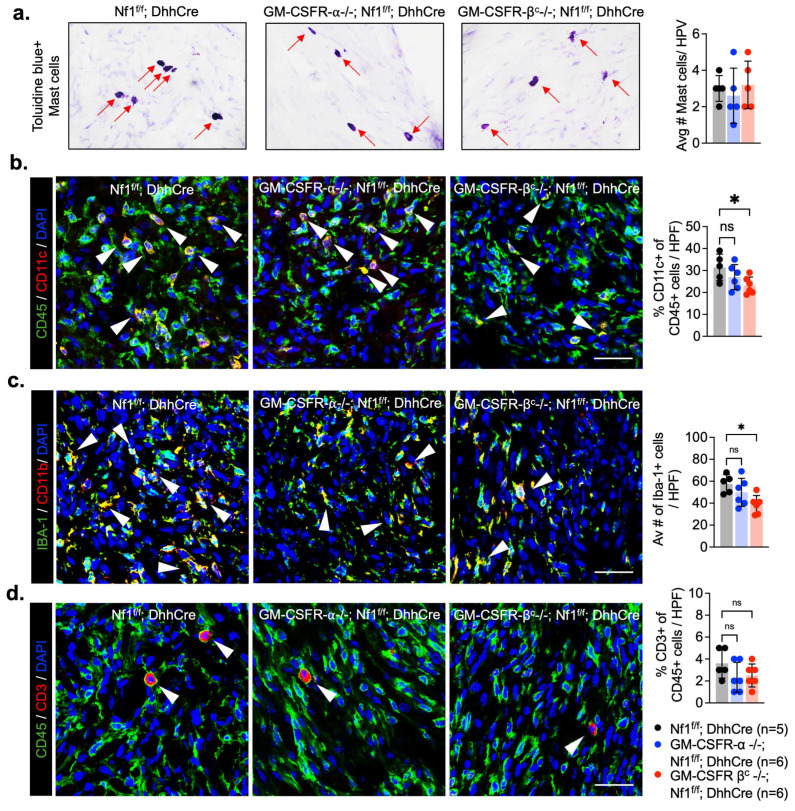
Loss of GM-CSFR-β^c^ reduced the presence of myeloid cells. Representative pictures of stained tumor tissue sections and their corresponding quantifications of (**a**) toluidine blue staining for mast cells (red arrows) (representative picture from each group, n = 4), (**b**) CD11c+ dendritic cells, (**c**) Iba – 1+ macrophages and (**d**) CD3+ T cells (white arrows indicate immune cell) (representative picture from tumor tissue of PNF control (n = 5) and each GM-CSFR receptor knockout (n = 6) mice). Two-way ANOVA multiple comparison test, * *p* < 0.05.

Given the abundance of inflammatory cells (largely macrophages and dendritic cells) in PNF [34,35], and that GM-CSF signaling is known to modulate the tumor-immune microenvironment in many settings [7], we analyzed tissues sections to quantify the types of immune cells in the mouse PNF lacking Csf2a or Csfr2b (Figure 3). Numbers of mast cells, identified as metachromatic (purple) toluidine blue positive cells per high powered field, did not differ among the GM-CSFR-α-/-; Nf1^f/f^; DhhCre (n = 6), GM-CSFR-β^c^-/-; Nf1^f/f^; DhhCre (n = 6), and Nf1^f/f^; DhhCre control (n = 5) groups (Figure 3a). In contrast, CD11c+ dendritic cells (Figure 3b) and Iba1+ macrophages (Figure 3c) were significantly reduced in sections from GM-CSFR-β^c^-/-; Nf1^f/f^; DhhCre PNF but not GM-CSFR-α-/-; Nf1^f/f^; DhhCre PNF. CD3+ T cell numbers remained unchanged (Figure 3d). Thus, a reduction in macrophages and DCs correlated with a reduced tumor number in GM-CSFR-β^c^-/-; Nf1^f/f^; DhhCre mutants.

### 3.4. Loss of GM-CSF Receptors Alters the PNF Inflammatory Proteome

**Figure 4:**
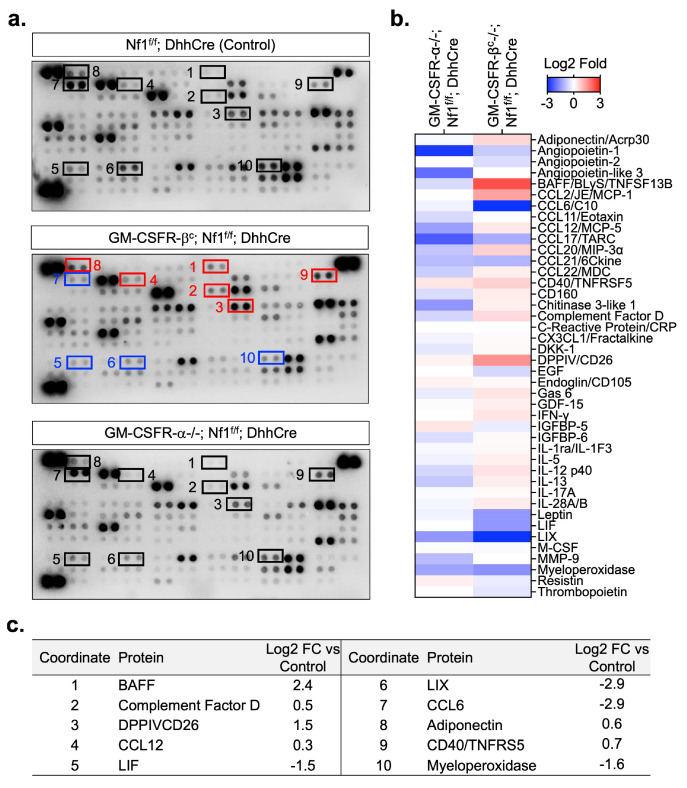
Altered proteome profile results from lack of GM-CSFR-α and GM-CSFR-β^c^. (**a**) Images of scanned proteome microarray blots incubated with lysates of tumors from Nf1^f/f^; DhhCre (control) and with GM-CSFR-α or GM-CSFR-β^c^ deletion. (**b**) Heatmap representation of proteome profile. (**c**) Cytokines that robustly increased or decreased after the loss of the GM-CSFR-β^c^ receptor.

We considered the possibility that changes in the cytokine milieu contribute to a reduced tumor number in GM-CSFR-β^c^-/-; Nf1^f/f^; DhhCre mutants and/or provide clues to mechanisms that compensate for the absence of GM-CSFR-β^c^ in PNF cells. To identify proteins that might contribute to the response or to the maintenance of immune cells, tumors from GM-CSFR-α-/-; Nf1^f/f^; DhhCre, GM-CSFR-β^c^-/-; Nf1^f/f^; DhhCre and Nf1^f/f^; DhhCre controls were lysed and probed individually using cytokine protein arrays. Figure 4a shows representative exposures. Signal intensities from each blot were visualized using heatmaps (Figure 4b). While this analysis is semi-quantitative and needs further validation, quantification based on scanning of blots (Figure 4c and Supplemental Figure S3) demonstrated robust changes, including increases in BAFF, Complement factor D, CD26, Adiponectin, and CD40 in GM-CSFR-β^c^-/-; Nf1^f/f^; DhhCre versus control tumor lysates. Some or all of these factors may contribute to the maintenance of a proinflammatory environment and to neurofibroma growth after loss of GM-CSFR-β^c^ receptors. We did not detect a compensatory increase in GM-CSF. The ligand IL5 was not significantly changed. Thus, loss of the βc receptor does not promote significant compensatory upregulation of this ligand, which also uses GM-CSFR-β^c^ for signaling; the possibility remains that IL5 or Il5ra has a role to play in these tumors. We conclude that GM-CSFR-β^c^ deletion causes compensatory changes in signaling pathways.

## 4. Discussion

Our results showed that at the transcriptional level, Csf2ra (GM-CSFR-α) and Csf2rb (GM-CSFR-β^c^) are expressed in tumor immune cells. Csfr2a is increased in tumor DC and macrophage subsets versus normal nerves. The majority of GM-CSFR-α+ protein expression is in myeloid cells, including Xcr1+ CD11c+ cDC1, SIRPα+ CD11c+ cDC2 dendritic cells and CD11b+; CD11c- macrophages. Expression of GM-CSFR-β^c^ was limited to fewer cells and did not differ between control and PNF. Thus, in neurofibroma, GM-CSFR-α and GM-CSFR-β^c^ are expressed predominantly by hematopoietic cells and myeloid cells, as in other tissues [40,44,45]. Based on the observed expression pattern, we anticipated that deletion of the GM-CSFR-α receptor subunit might substantially reduce the number of tumor myeloid cells. However, loss of GM-CSFR-α did not affect the number of myeloid immune cells or reduce the number of tumors. Rather, the loss of GM-CSFR-β^c^ reduced the number of tumors and the numbers of CD11c+ tumor dendritic cells and tumor macrophages. These results are consistent with the known function of GM-CSFR-β^c^ in regulating macrophages and DCs [51,52]. The loss of neither receptor altered the numbers of mast cells and T cells; the remaining T cells were likely to continue to play a critical role in promoting PNF development [42]. 

The finding that GM-CSFR-β^c^ contributes to PNF formation is consistent with roles for this receptor in tissue pathology, for example, in hematopoietic and pulmonary settings [40,44]. Our data are also consistent with a role for GM-CSFR-β^c^ in NF1-associated JMML, and a contribution of GM-CSFR-β^c^ to skin hyper-pigmentation after nerve injury [23,26,38]. In contrast, in JMML, deletion of GM-CSF itself phenocopied the deletion of β^c^, while in the neurofibroma model tested here, deletion of GM-CSFR-α (the specific subunit that binds GM-CSF) did not phenocopy the loss of β^c^. Thus, the effects of GM-CSFR-β^c^ in PNF are not mediated through GMCSF. It is more likely that GM-CSFR-β^c^ is activated through an alternative ligand and an alternative α subunit. Alpha (α) subunits bind β^c^ to transduce signals from Interleukin 3 (IL-3) and Interleukin 5 (IL-5) [6,51,53]. While neither of these interleukins has been studied in NF1, in other tissues, as with GM-CSF, they are involved in inflammatory responses, including immune cell homeostasis and tumor pathology [54]. Consistent with the usage of alternative ligands, our cytokine arrays demonstrate sustained expression of IL-5, a T cell cytokine, in tumors with loss of GM-CSFR-β^c^. Notably, MEK inhibition reduced expression of IL5 in neurofibroma lysates, correlating with reduced tumor growth [55]. In a RAS-driven model of chronic pancreatitis and pancreatic tumor formation, IL5 expression increased in early lesions, followed by increased IL5R-α expression [56], consistent with the idea that the β-common ligand IL5 can play roles in solid tumors, which requires further study. IL5 is largely known for its effects on eosinophils and B cells [2]; neither of these cell types has been described in neurofibromas. A decrease in the T cell and DC chemoattractant CXCL10 after GM-CSFR-β^c^ deletion is consistent with the role of CXCL10–CXCR3 pathway in driving PNF formation [43]. This reduction may in part explain the effects of β^c^ deletion on reducing neurofibroma formation. Expression of other factors increased in response to GM-CSFR-β^c^ deletion; these included B-cell activation factor (BAFF), CCL2, CCL12, CD26, and Adiponectin, which may be involved in compensatory pathways.

We note that loss of GM-CSFR-β^c^ was dispensable for the Remak bundle disruption in PNF, a neuropathology observed in human tumors as well as mouse models [29,46,47,48,49]. Further work is needed to study the role of cytokines modulated by GM-CSFR-β^c^ loss in PNF formation.

## 5. Conclusions

In conclusion, the absence of GM-CSFR-βc significantly reduces the number of peripheral nerve tumors and decreases the accumulation of key immune cells like macrophages and dendritic cells. This change indicates a shift in the cytokine and chemokine profile within PNFs, suggesting that factors beyond GM-CSF contribute to neurofibroma formation. This underscores the importance of exploring alternative pathways for effective treatments.

## Data Availability

Data obtained for the single cell-RNA seq is available in the NCBI Gene Expression Omnibus database (GEO GSE181985).

## Supplementary Materials

The following supporting information can be downloaded at: https://www.mdpi.com/article/10.3390/cancers17050905/s1, Figure S1. Schematic illustration of breeding strategy. Homozygous mutant, GM-CSFRa-/- (Csf2ra-/-) and GM-CSFRbc-/- (Csf2rb-/-) mice were crossed with Nf1f/f or Nf1f/+; DhhCre mice to generate either GM-CSFRa-/-; Nf1f/f; DhhCre and GM- CSFRbc-/-; Nf1f/f; DhhCre mice after several crosses; Figure S2. Representative images of a hematoxylin and eosin (H&E) stain of lung tissues taken from 7-month old Nf1f/f; DhhCre (control), GM-CSFRa-/-; Nf1f/f; DhhCre or GM-CSFRbc-/-; Nf1f/f; DhhCre mice. Figure S3. A table showing the 2-fold change in signal intensity of cytokines detected on a cytokine array from tumor lysates. The samples were taken from three groups of mice: GM-CSFRα-/-; Nf1f/f; DhhCre, GM-CSFRβc-/-; Nf1f/f; DhhCre, and Nf1f/f; DhhCre (control).
