# Supplementary material for: Granulocyte-Macrophage Colony Stimulating Factor Receptor Contributes to Plexiform Neurofibroma Initiation
Source: Cancers (Basel). 2025 Mar 6;17(5):905. doi: 10.3390/cancers17050905 (PMC11899227; doi:10.3390/cancers17050905)
Supplement: Supplementary file 1 [file cancers-17-00905-s001.zip › cancers-3412738-supplementary.pdf]

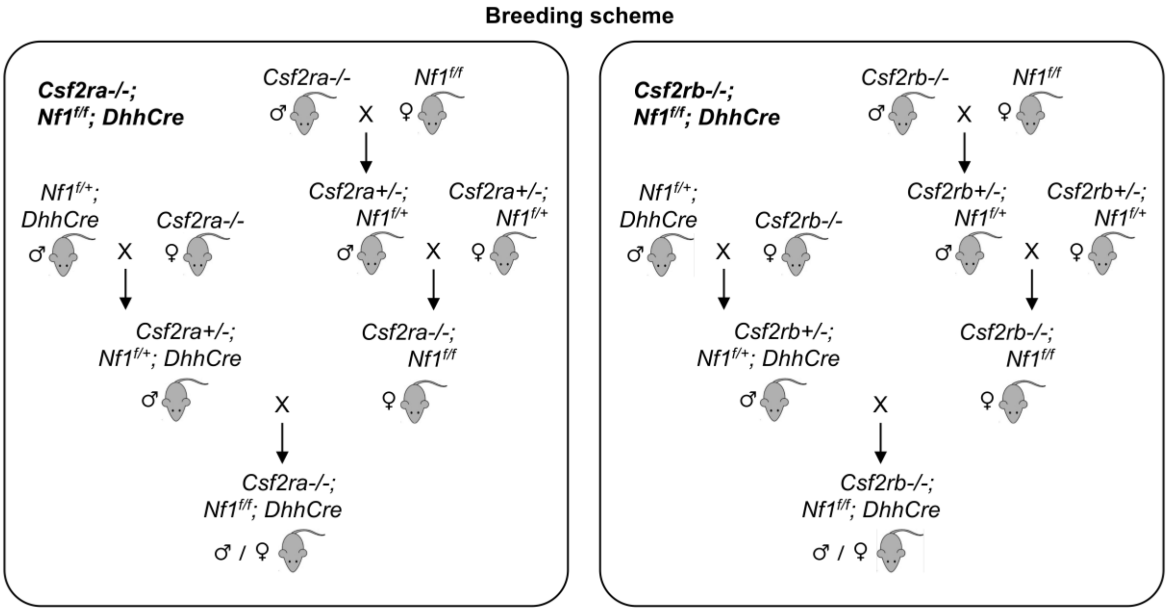

Supplemental Figure S1. Schematic illustration of breeding strategy. Homozygous mutant, GM-CSFRa-/- (Csf2ra-/-) and GM-CSFRbc-/- (Csf2rb-/-) mice were crossed with Nf1f/f or Nf1f/+; DhhCre mice to generate either GM-CSFRa-/-; Nf1f/f; DhhCre and GM-CSFRbc-/-; Nf1f/f; DhhCre mice after several crosses.

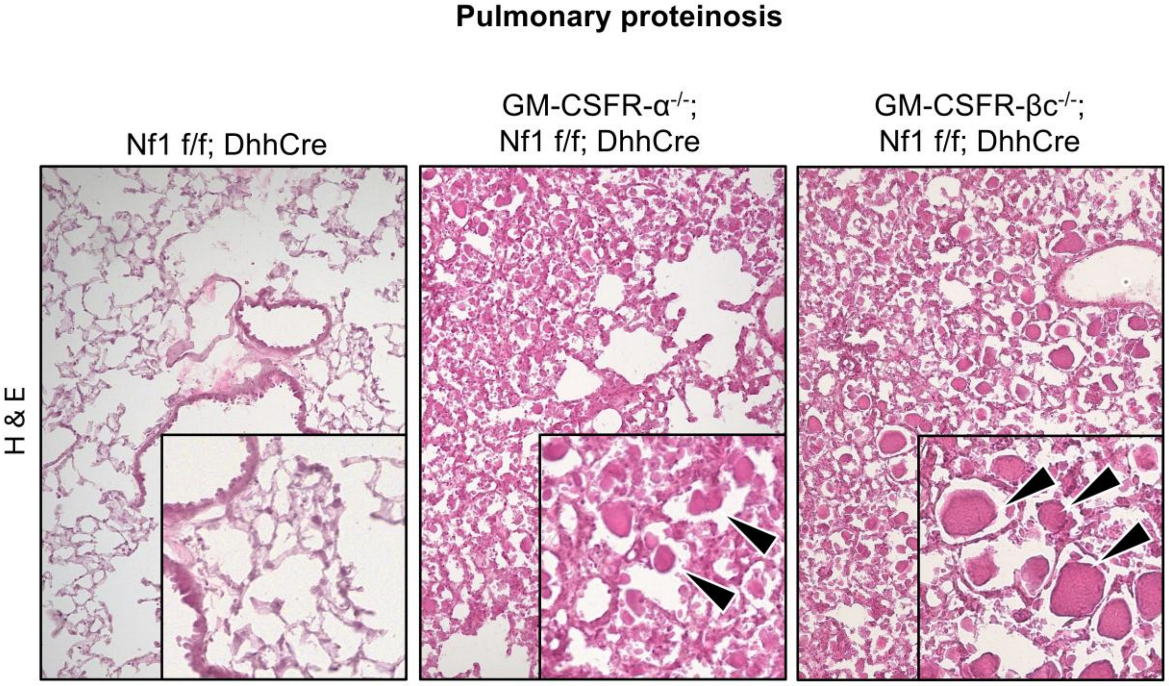

Supplemental Figure S2. Representative images of a hematoxylin and eosin (H&E) stain of lung tissues taken from 7-month old Nf1f/f; DhhCre (control), GM-CSFRa-/-; Nf1f/f; DhhCre or GM-CSFRbc-/-; Nf1f/f; DhhCre mice.

Cytokines	Control (Nf1f/f; DhhCre)	GM-CSFR-α-/-; Nf1f/f; DhhCre	GM-CSFR-βc-/-; Nf1f/f; DhhCre
Adiponectin/Acrp30	0.00	-0.11	0.58
Angiopoietin-1	0.00	-2.80	-0.80
Angiopoietin-2	0.00	-3.40	-0.50
Angiopoietin-like 3	0.00	-2.08	0.01
BAFF/BLyS/TNFSF13B	0.00	-0.56	2.35
CCL2/JE/MCP-1	0.00	-0.02	1.32
CCL6/C10	0.00	-0.20	-2.90
CCL11/Eotaxin	0.00	-0.59	0.00
CCL12/MCP-5	0.00	-1.40	0.33
CCL17/TARC	0.00	-2.30	-1.20
CCL20/MIP-3α	0.00	-0.90	0.69
CCL21/6Ckine	0.00	-1.00	-1.10
CCL22/MDC	0.00	-0.74	0.25
CD160	0.00	-0.55	0.27
Chitinase 3-like 1	0.00	-1.55	0.22
Complement Factor D	0.00	-0.61	0.55
C-Reactive Protein/CRP	0.00	-0.01	0.00
CX3CL1/Fractalkine	0.00	-0.18	0.08
DKK-1	0.00	-0.38	0.09
DPPIV/CD26	0.00	0.17	1.54
EGF	0.00	-3.30	-0.60
Gas 6	0.00	-0.31	0.39
GDF-15	0.00	-0.05	0.25
IFN-γ	0.00	-0.01	0.39
IGFBP-5	0.00	0.37	-0.30
IGFBP-6	0.00	-0.54	0.09
IL-1ra/IL-1F3	0.00	-0.11	0.11
IL-5	0.00	-0.21	0.22
IL-12 p40	0.00	-0.61	0.41
IL-13	0.00	-0.84	0.27
IL-17A	0.00	-0.09	0.09
IL-28A/B	0.00	-0.15	0.29
Leptin	0.00	-0.20	-1.50
LIF	0.00	0.01	-1.50
LIX	0.00	-1.50	-2.90
M-CSF	0.00	0.02	-0.10
MMP-9	0.00	-1.00	-3.30
Resistin	0.00	0.23	-0.30
Thrombopoietin	0.00	0.04	-0.40

Supplemental Figure S3. A table showing the 2-fold change in signal intensity of cytokines detected on a cytokine array from tumor lysates. The samples were taken from three groups of mice: GM-CSFRα-/-; Nf1f/f; DhhCre, GM-CSFRβc-/-; Nf1f/f; DhhCre, and Nf1f/f; DhhCre (control).
